# *S*-nitrosocysteamine-functionalised porous graphene oxide nanosheets as nitric oxide delivery vehicles for cardiovascular applications

**DOI:** 10.1016/j.redox.2024.103144

**Published:** 2024-04-04

**Authors:** Tanveer A. Tabish, Mian Zahid Hussain, Sevasti Zervou, William K. Myers, Weiming Tu, Jiabao Xu, Irina Beer, Wei E. Huang, Rona Chandrawati, Mark J. Crabtree, Paul G. Winyard, Craig A. Lygate

**Affiliations:** a Division of Cardiovascular Medicine, Radcliffe Department of Medicine, British Heart Foundation (BHF) Centre of Research Excellence, University of Oxford, Headington, Oxford, OX3 7BN, United Kingdom; b School of Natural Sciences and Catalysis Research Centre, Department of Chemistry, Chair of Inorganic and Metal-Organic Chemistry, Technical University of Munich (TUM), Lichtenbergstraße 4, 85748, Garching, Germany; c Centre for Advanced Electron Spin Resonance (CAESR), Inorganic Chemistry Laboratory, Department of Chemistry, University of Oxford, Oxford, OX1 3QR, United Kingdom; d Department of Engineering Science, University of Oxford, Oxford, OX1 3PJ, United Kingdom; e James Watt School of Engineering, University of Glasgow, Glasgow, G12 8QQ, United Kingdom; f Institute of Water Chemistry, Chair of Analytical Chemistry and Water Chemistry, Technical University of Munich, Lichtenbergstraße 4, 85748, Garching, Germany; g School of Chemical Engineering and Australian Centre for Nanomedicine (ACN), The University of New South Wales (UNSW), Sydney, NSW, 2052, Australia; h Department of Biochemical Sciences, School of Biosciences and Medicine, University of Surrey, Guildford, GU2 7XH, United Kingdom; i University of Exeter Medical School, Faculty of Health and Life Sciences, University of Exeter, Exeter, EX1 2LU, United Kingdom

## Abstract

Nitric oxide (NO) is a key signalling molecule released by vascular endothelial cells that is essential for vascular health. Low NO bioactivity is associated with cardiovascular diseases, such as hypertension, atherosclerosis, and heart failure and NO donors are a mainstay of drug treatment. However, many NO donors are associated with the development of tolerance and adverse effects, so new formulations for controlled and targeted release of NO would be advantageous. Herein, we describe the design and characterisation of a novel NO delivery system via the reaction of acidified sodium nitrite with thiol groups that had been introduced by cysteamine conjugation to porous graphene oxide nanosheets, thereby generating *S*-nitrosated nanosheets. An NO electrode, ozone-based chemiluminescence and electron paramagnetic resonance spectroscopy were used to measure NO released from various graphene formulations, which was sustained at >5 × 10^−10^ mol cm^−2^ min^−1^ for at least 3 h, compared with healthy endothelium (cf. 0.5–4 × 10^−10^ mol cm^−2^ min^−1^). Single cell Raman micro-spectroscopy showed that vascular endothelial and smooth muscle cells (SMCs) took up graphene nanostructures, with intracellular NO release detected via a fluorescent NO-specific probe. Functionalised graphene had a dose-dependent effect to promote proliferation in endothelial cells and to inhibit growth in SMCs, which was associated with cGMP release indicating intracellular activation of canonical NO signalling. Chemiluminescence detected negligible production of toxic *N*-nitrosamines. Our findings demonstrate the utility of porous graphene oxide as a NO delivery vehicle to release physiologically relevant amounts of NO *in vitro*, thereby highlighting the potential of these formulations as a strategy for the treatment of cardiovascular diseases.

## 1 Introduction

Cardiovascular disease remains the leading cause of morbidity and mortality worldwide and is associated with endothelial dysfunction [1]. The endothelium is a monolayer of endothelial cells (ECs) that lines the inner surface of blood vessels and releases intercellular signalling molecules such as nitric oxide (NO) to promote vascular health and vasodilation [2,3]. ECs produce NO continuously through the enzymatic conversion of *l*-arginine and molecular oxygen to NO and *l*-citrulline catalysed by endothelial NO synthase (eNOS) [4]. NO is also important in response to injury, since it inhibits the activation of platelets and inflammatory cells and promotes EC proliferation, while inhibiting over-proliferation of the underlying layer of smooth muscle cells (SMCs) [5]. For these reasons, the exogenous administration of NO in the form of organic nitrates, nitrites and inorganic (metal nitrosyl) compounds has remained a cornerstone of cardiovascular pharmacology for more than a century [6,7]. However, there are a number of limitations of such compounds, including chemical instability, nitrate tolerance and common adverse effects, such as headache, which limit their therapeutic utility [8].

Over the past decade, nanoparticles (NPs) have been designed to store and release NO in a controlled manner [9]. Incorporation of various NO donors such as small diazeniumdiolate molecules, *S*-nitrosothiols (RSNOs), and NO gas into different classes of NPs have been reported in the literature, e.g. silica NPs [10], dendrimers [11,12], gold NPs [13,14], hydrogel NPs [15], and other polymeric NPs [16,17]. Often these have been designed for wound healing or to prevent biofilms forming on medical devices. Cardiovascular applications have included the NO donor, *S*-nitroso-*N*-acetylpenicillamine (SNAP) conjugated to dendrimers to reduce myocardial ischaemia/reperfusion injury [18,19], and liposome NPs encapsulating NO gas to reduce neointimal hyperplasia (SMC proliferation) in a rabbit model of vascular injury [20]. Current issues include instability leading to spontaneous NO release under physiologically conditions, non-specific NO delivery due to leaching of NO donors and generation of toxic chemicals such as *N*-nitrosoamines.

Graphene nanostructures have received increasing attention in recent years since their physicochemical properties can be fine-tuned to optimise storage and release of drugs and biomolecules. Graphene oxide (GO) is an oxygen-rich form of graphene formed by strong oxidation and containing hydroxyl groups at the basal plane and carboxyl groups at the edges. Porous graphene oxide (PGO) has the same functional groups, but pores on the basal planes and between the stacked sheets of graphene [21], which confers unique properties including high porosity, specific surface area, and electron mobility in comparison to pure graphene [22,23]. We hypothesised that this highly porous structure, enhanced distance between graphene layers, and high reactivity would provide greater storage capability for NO sources.

Here we report the design of *S*-nitrosocysteamine-functionalised porous graphene oxide (termed SNO-Cys@PGO) for the controlled release of NO. Acidified sodium nitrite was used as the source of NO since this mixture generates reactive nitrogen species which *S*-nitrosate thiols to form *S*-nitrosothiols. Subsequent release of NO from RSNOs occurs as a result of the relatively low RS-NO bond energies, which typically fall within the range of 20–32 kcal mol^−1^ [24,25]. We show that intracellular release of NO from functionalised formulations of GO significantly enhances endothelial cell proliferation and inhibits smooth muscle cell growth in a concentration dependent manner *in vitro*. This provides proof-of-concept that a novel *S-*nitrosothiol-functionalised form of porous graphene can be engineered to achieve controlled and physiologically relevant release of NO, with potential for cardiovascular applications.

## 2 Results and discussion

### 2.1 Design, synthesis, and optimisation of functionalised graphene conjugates

**Fig. 1:**
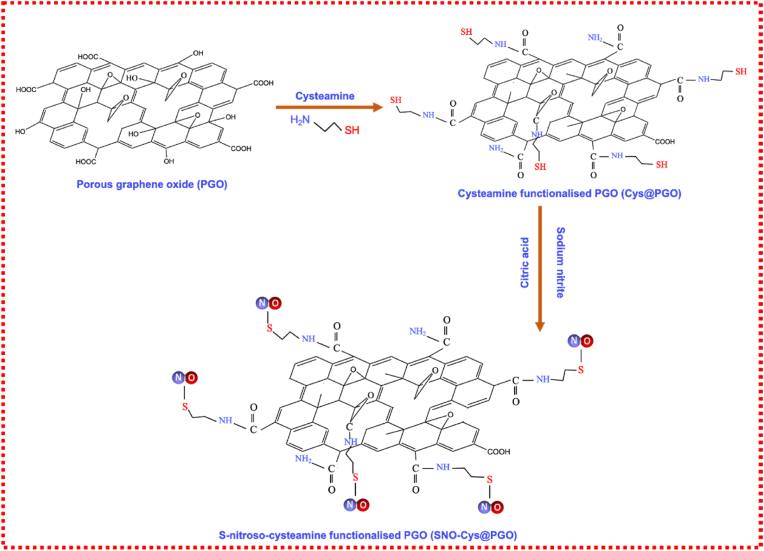
A schematic illustration of the crosslinking of cysteamine to porous graphene oxide (PGO) followed by the *S*-nitrosation of cysteamine-functionalised PGO to form *S*-nitrosocysteamine-functionalised PGO (termed SNO-Cys@PGO).

GO was synthesised using a modified Hummer's method, and the detailed synthesis and characterisation methods are described in our previous publications [[26], [27], [28], [29]]. In this work, we also describe a scalable, cost-effective and a binder-free method of preparing PGO via one-step acid activation that allows a tuneable and uniform pore diameter distribution and controllable morphology. Nitric acid (HNO_3_) was used to react with the oxygen-containing defect clusters that exist on GO sheets. It acts as a dehydrating agent in an inert atmosphere at room temperature, involving a one-step sonication, which results in the removal of oxygenated carbon regions and the introduction of in-plane porosity on GO sheets [30]. Based on the carboxyl functional groups, GO and PGO were modified with cysteamine to introduce thiol groups followed by the nitrosation of the thiol groups to provide *S*-nitroso groups capable of NO release. A schematic illustration of the conjugation is shown for PGO in Fig. 1 and for GO in SI Fig. 1.

**Fig. 2:**
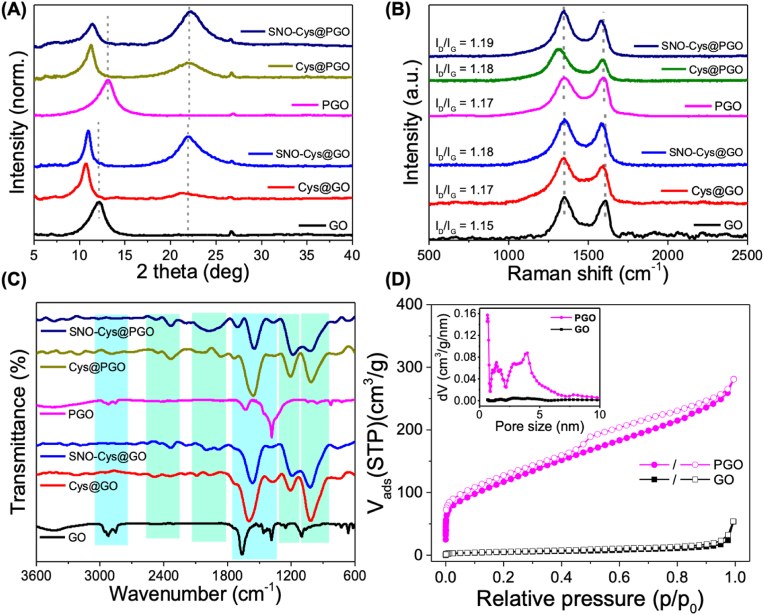
Characterisation of GO, Cys@GO, SNO-Cys@ GO, PGO, Cys@PGO and SNO-Cys@ PGO. (**A**) X-ray diffraction (XRD) patterns, (**B**) Raman spectra, (**C**) FTIR where blue highlighted peaks correspond to GO/PGO, and green highlighted peaks represent conjugated cysteamine/acidified sodium nitrite. (**D**) N_2_ isotherms of GO and PGO. The solid and open symbols show adsorption and desorption isotherms, respectively. The pore size distribution of PGO (inset) after activation with HNO_3_, which was characterised by NLDFT method. Compared to the GO, the PGO exhibits the manifold increase in micro/mesopores between 1 and 15 nm. (For interpretation of the references to colour in this figure legend, the reader is referred to the Web version of this article.)

Functionalised GO and PGO was characterised by X-ray diffraction (XRD), Fourier transform infrared spectroscopy (FTIR), Raman spectroscopy and scanning electron microscopy (SEM) at each synthesis step to shed light on the mechanism of *S*-nitrosation of cysteamine-functionalised GO and PGO. The XRD patterns for GO and PGO were similar to the typical sp^2^-hybridised carbon, with peaks at 2θ = 11.97° and 12.92° respectively, indicating the presence of a crystalline graphene structure with interlayer spacing of 7.4 and 6.9 Å respectively (Fig. 2 A). A slight compression in interlayer spacing of PGO compared to the GO, is due to the partial removal of oxygenated carbon and oxygen containing defect clusters upon HNO_3_ treatment, which is inevitable for porosity generation. The thiolation of GO and PGO shows a significant shift in peaks at 2θ from 11.97° to 10.62° (GO) and from 12.92° to 11.12° (PGO) respectively, corresponding to a wider interlayer spacing of 8.3 Å for GO and 8.0 Å for PGO. The extension in interlayer spacing is due to the strong interaction between the thiol group of cysteamine intercalated into GO and PGO. Interestingly, one additional peak around 2θ = 22° was observed in both thiolated samples of GO and PGO, which became more prominent after adding acidified sodium nitrite. This partial compression in interlayer spacing suggests a successful reaction of *S*-nitrosating species (reactive nitrogen species generated from the acidification of sodium nitrite) with thiolated GO and PGO through covalent bonding, formation of high amount of defects in graphene [31] as well as the presence of amide groups [32] and the process of thiolation [33], thus confirming the successful conjugation of GO and PGO with thiol groups and *S*-nitrosation. The typical XRD peaks and the corresponding groups of cysteamine and acidified sodium nitrite can be seen in SI Fig. 2. Two typical Raman bands are found in graphene and functionalised graphene samples, with the D band at 1350 cm^−1^ representative of oxidising or edge defects in carbon sp^2^ atoms and G band at 1600 cm^−1^ assigned to pristine sp^2^ graphitic layer (Fig. 2B). The intensity ratio between D and G bands (I_D_/I_G_) is typically used to examine the defective nature associated with the degree of functionalisation of sp^2^ hybridised carbon [34]. The intensity ratio of D and G peak was found to be larger in PGO than GO due to the activation of GO with HNO_3_. The higher degree of thiol groups attached to GO and PGO, increases the I_D_/I_G_ ratio, evidencing effective thiol functionalisation associated with defects of the graphitic planes. Furthermore, for the *S*-nitrosation of cysteamine-functionalised GO and PGO, the ratio I_D_/I_G_ increases further indicating that acidified nitrite produced a covalent modification of graphene. The typical Raman peaks and the corresponding groups of cysteamine and acidified sodium nitrite can be seen in SI Fig. 3. In order to evaluate the successful functionalisation of GO and PGO, FTIR spectroscopy was used. The FTIR spectrum for GO showed typical bands of O–H (2924 cm^−1^) and CO (1745 cm^−1^) stretching due to oxygenated groups. The bands at 1384, and 1098 cm^−1^ represent the carboxyl and epoxy groups, respectively. Upon covalent attachment of cysteamine to graphene, Cys@GO and Cys@PGO presented several bands at S–H stretching (2341 cm^−1^), amide CO stretching (1600 cm^−1^), N–H bending (3292 cm^−1^), C–N stretching (1384 cm^−1^) and N–H wagging (725 cm^−1^) related to the amide bonds and thiol groups. The peak at 1660 cm^−1^ corresponds to the presence of NH_2_ in plane bending. The peak at 1209 cm^−1^ is due to the presence of *C*–N–H scissoring (Fig. 2C). A small difference was observed when comparing the FTIR spectra of cysteamine-functionalised and *S*-nitrosocysteamine-functionalised graphene samples, showing the interaction between cysteamine functionalised graphene and acidified sodium nitrite. There is a sharp difference in peak intensity at 725 and 1209 cm^−1^ which shows *S*-nitrosation on the surface of both GO and PGO. The typical FTIR peaks and the corresponding groups of cysteamine and acidified sodium nitrite can be seen in SI Fig. 4. To examine the porous morphology of the PGO, the N_2_ sorption isotherms were assessed. The calculated Brunauer–Emmett–Teller (BET) specific surface areas and the total pore volumes of the as-prepared PGO were significantly higher than GO (Fig. 2 D). The GO sample exhibited a BET surface area of 68 m^2^ g^-1^ while PGO had a surface area of 424 m^2^ g^-1^ and total pore volume of 0.43 cm^3^ g^-1^, with a more prominent pore size distribution in the micro/mesoporous range of 1–5 nm (the inset figure is also given in (SI Fig. 5). This demonstrates the existence of small mesopores in the basal plane of PGO and validates the successful formation of porous graphene oxide.

**Fig. 3:**
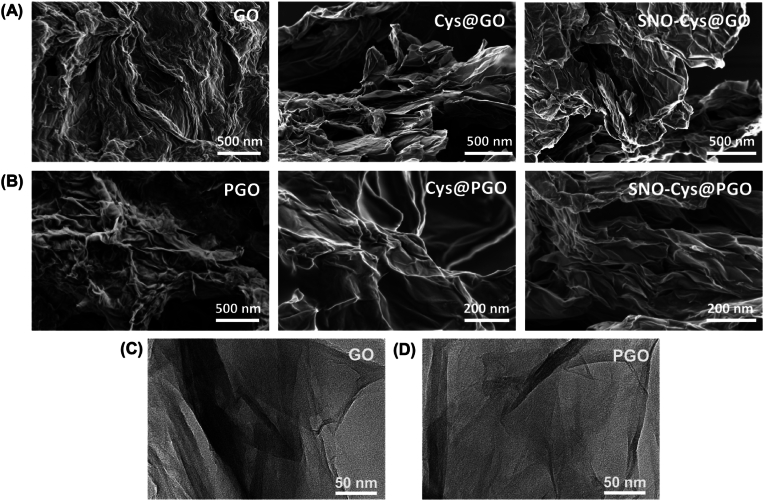
**Morphological characterisation of graphene oxide (GO) and porous graphene oxide (PGO).** Representative SEM images of (**A**) GO, Cys@GO, and SNO-Cys@GO, and (**B**) PGO, Cys@PGO, and SNO-Cys@PGO. (**C, D**) Representative TEM images of GO and PGO.

Representative SEM micrographs are shown in Fig. 3A for GO, Cys@GO, and SNO-Cys@GO, samples, while Fig. 3B shows the same progression for PGO, Cys@PGO and SNO-Cys@PGO. These images indicate an interconnected network of randomly oriented sheets, with the functionalised versions of GO and PGO (i.e. Cys@GO, Cys@PGO, SNO-Cys@GO and SNO-Cys@PGO) presenting with more stacked sheets because of thiolation and functionalisation. Fig. 3C and 3D shows representative TEM images for the pristine GO and PGO sheets, revealing the interconnected networks of sheet-like structure of graphene.

**Fig. 4:**
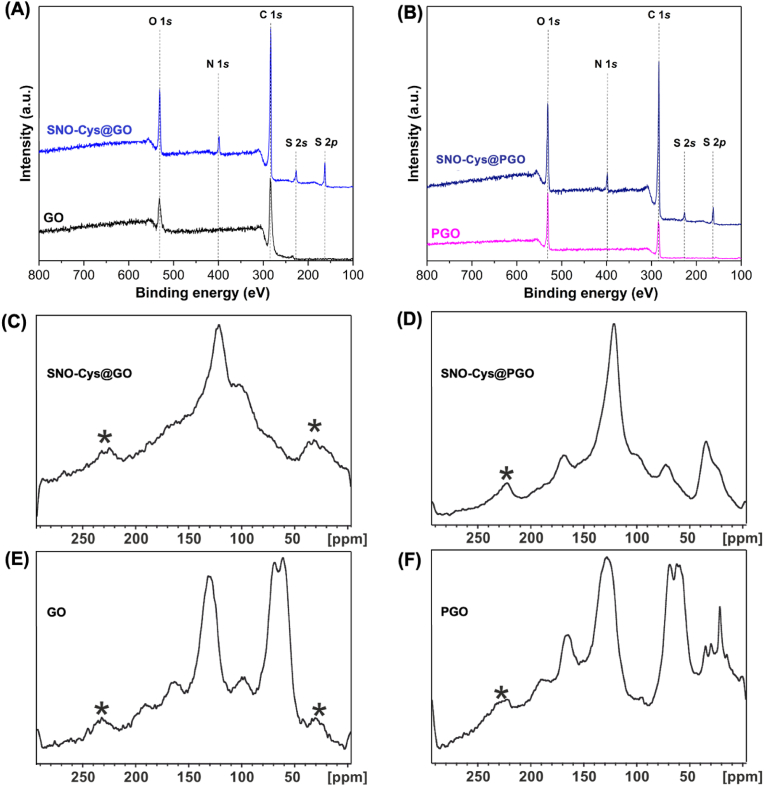
**XPS and NMR characterisation of graphene formulations. (A**–**B)** XPS survey spectra of GO, SNO-Cys@GO, PGO, and SNO-Cys@PGO. **(C**–**F)**^13^C SSNMR spectra of GO, SNO-Cys@GO, PGO, and SNO-Cys@PGO measured at a 10 kHz Magical Angle Spinning (MAS) rate. (*) Indicates spinning side bands, i.e. spurious signals caused by magnetic field modulation at the spinning frequency, which typically appear at a separation equal to the spinning rate on either side of the authentic high intensity peaks of the samples.

The X-ray photoelectron spectroscopy (XPS) survey spectra of GO, SNO-Cys@GO, PGO, and SNO-Cys@PGO were examined to determine the chemical composition of the samples. The survey spectra of GO and PGO confirmed the presence of C and O. In addition, Fig. 4 A-B shows the peak of O 1s having a binding energy of 530.5 eV, which is in good agreement with the binding energy for GO and PGO in the literature [35,36]. The functionalisation of GO and PGO with cysteamine was confirmed by the presence of S (2s and 2p) and with nitrite by the presence of N. The presence of these elements confirms the successful conjugation of GO and PGO with cysteamine and the successful *S*-nitrosation of the free thiols provided by cysteamine conjugation. Additionally, the absence of any other trace elements confirms that the samples were contamination free. Nuclear magnetic resonance (NMR) was used to examine the functional group formation and to confirm the successful conjugation of graphene with cysteamine and the subsequent *S*-nitrosation of the free thiols of the GO/PGO-conjugated cysteamine. ^13^C NMR spectra of GO (Fig. 4E) and PGO (Fig. 4F) showed similar peaks at 60, 70, and 130 ppm. The peaks at 60 and 70 ppm correspond to tertiary *C*–*O*–C and *C*–OH groups, respectively, whereas the peak at 130 ppm originates from graphitic sp^2^ carbon [37]. The less intense peaks around 165 and 190 ppm correspond to COOH and CO groups, respectively [38]. SNO-Cys@GO (Fig. 4C) and SNO-Cys@PGO (Fig. 4D) exhibited a shift at 41.2 ppm, which is attributed to the carbon next to the *S*-nitrosothiol group [39]. Solid state magic angle spinning (MAS) NMR spectra of GO, SNO-Cys@GO, PGO, and SNO-Cys@PGO measured at two different magic angle spinning (MAS) rates of 14 and 10 kHz are given in the supplementary information (SI Figs. 6–9).

### 2.2 NO release measurements

**Fig. 5:**
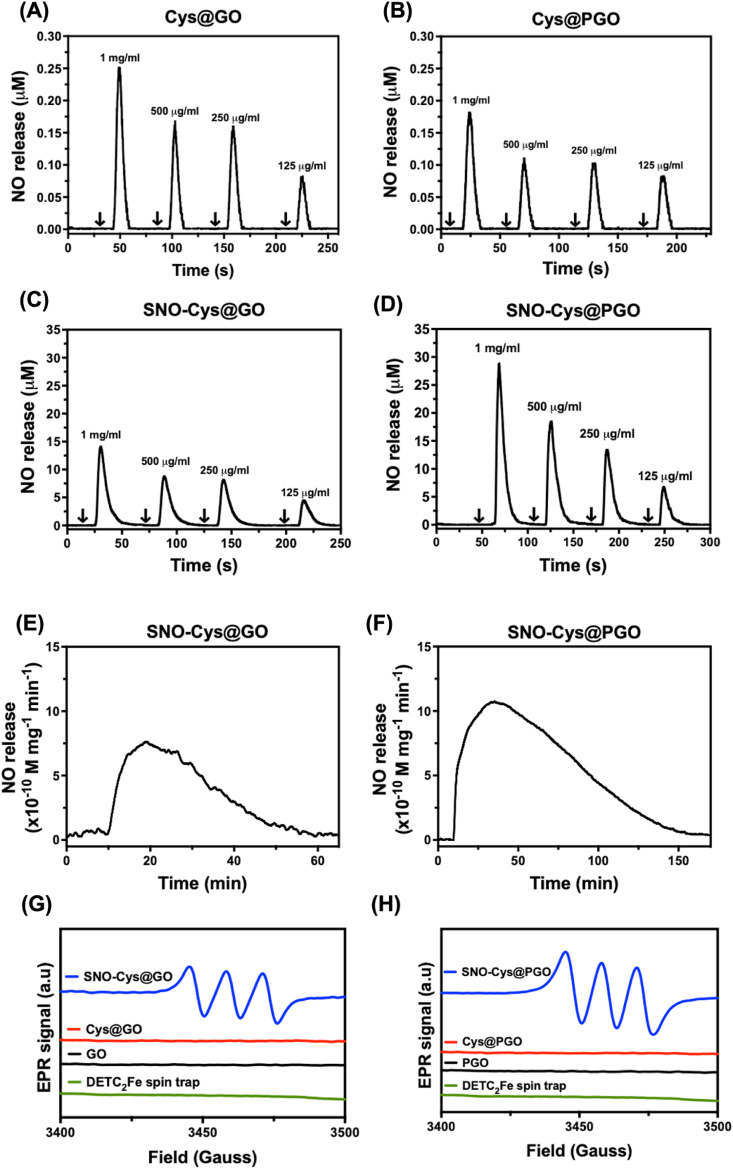
**Release of NO from different forms of graphene at pH 7.4 in 1*****X*****PBS.** Spontaneous NO release was evaluated using ozone-based chemiluminescence. Representative NO release from **(A)** Cys@GO, **(B)** Cys@PGO, **(C)** SNO-Cys@GO, **(D)** SNO-Cys@PGO. Arrow (↓) indicates the injection of graphene formulations at different concentrations, with x-axis showing the time duration since the start of the experimental run. **(*E***–**F)** Real-time NO release from graphene formulations at a concentration of 250 μg/ml using an electrochemical sensor at pH 7.4 in PBS. Representative NO release from **(E)** SNO-Cys@GO and **(F)** SNO-Cys@PGO. **(G**–**H)** NO release from different forms of graphene detected as a spin trap complex using electron paramagnetic resonance (EPR) spectrometry. X-band continuous wave (CW)-EPR spectra showing the extent of NO release from SNO-Cys@GO, Cys@GO, GO, SNO-Cys@PGO, Cys@PGO, PGO at a concentration of 250 μg/ml and DETC_2_Fe spin trap as detected by the formation of a NO–Fe(II)DETC_2_ complex (n = 3).

The total production of NO from graphene formulations was measured by ozone-based chemiluminescence (Fig. 5A–D). Fig. 5 A and B show that Cys@GO and Cys@PGO, without *S*-nitrosation, produce negligible amounts of NO (note the scale of the *y*-axis). Spontaneous NO release was from SNO-Cys@GO and SNO-Cys@PGO was observed as shown in Fig. 5C and D. The total NO production from SNO-Cys@PGO was much higher than SNO-Cys@GO and the NO production from SNO-Cys@GO and SNO-Cys@PGO occurred in a concentration-dependent manner. Maximal NO release was observed at 29 μM from SNO-Cys@PGO and 13 μM from SNO-Cys@GO. The real-time kinetics of NO release from both functionalised GO and PGO was investigated via a NO electrochemical sensor in phosphate-buffered saline (PBS), pH 7.4, at 37 °C under continuous stirring, which can selectively detect the production of NO above ≈1 nM [40]**.** NO release detected from SNO-Cys@GO was rapid over the first 10 min followed by slow and sustained release over 50 min as shown in Fig. 5E. In contrast, NO release from SNO-Cys@PGO was rapid over the first 25 min followed by slow and sustained release over 140 min as shown in Fig. 5F.

Electron paramagnetic resonance (EPR) spectroscopy was used to further confirm the release of NO from graphene derivatives by identifying the signature paramagnetic characteristics of NO, which are influenced by hyperfine splitting originating from the nitrogen nucleus of NO [41]. A (DETC)_2_Fe complex was used to trap NO released from samples, producing the EPR-active spin adduct, (DETC)_2_Fe–NO. The characteristic three-line isotropic EPR spectrum of (DETC)_2_Fe–NO revealed that NO gas was released from SNO-Cys@GO and SNO-Cys@PGO only (Fig. 5G and H), the spectrum having an isotropic g-value of 2.0035 (SNO-Cys@GO and SNO-Cys@PGO) and a ^14^N hyperfine coupling of 36 MHz or 0.5 mT. This signal was not detected in the samples of Cys@GO, GO, Cys@PGO, PGO or (DETC)_2_Fe spin trap complex. Taken together, these results demonstrate the prospect of designing functionalised graphene to store and release NO by tailoring the porosity and surface area.

### 2.3 Endothelial cell proliferation

The effect of graphene formulations on the viability and proliferation of cultured sEND.1 murine endothelial cells was investigated over a 24-h period. The MTT assay is a colorimetric assay that detects metabolic activity via the intracellular reduction of 3-(4,5-dimethylthiazol-2-yl)-2,5-diphenyltetrazolium bromide to a visible formazan dye, which is proportional to the number of live cells, i.e. it reports on cell viability and proliferation. Bromo-2-deoxyuridine (BrdU) was used to specifically evaluate cell proliferation, since BrdU is taken up and incorporated into newly synthesised DNA, the signal is proportional to the number of newly formed cells only. The lactate dehydrogenase (LDH) release assay is a cytotoxicity assay used to evaluate the level of plasma membrane damage by measuring the release of the intracellular enzyme LDH.

**Fig. 6:**
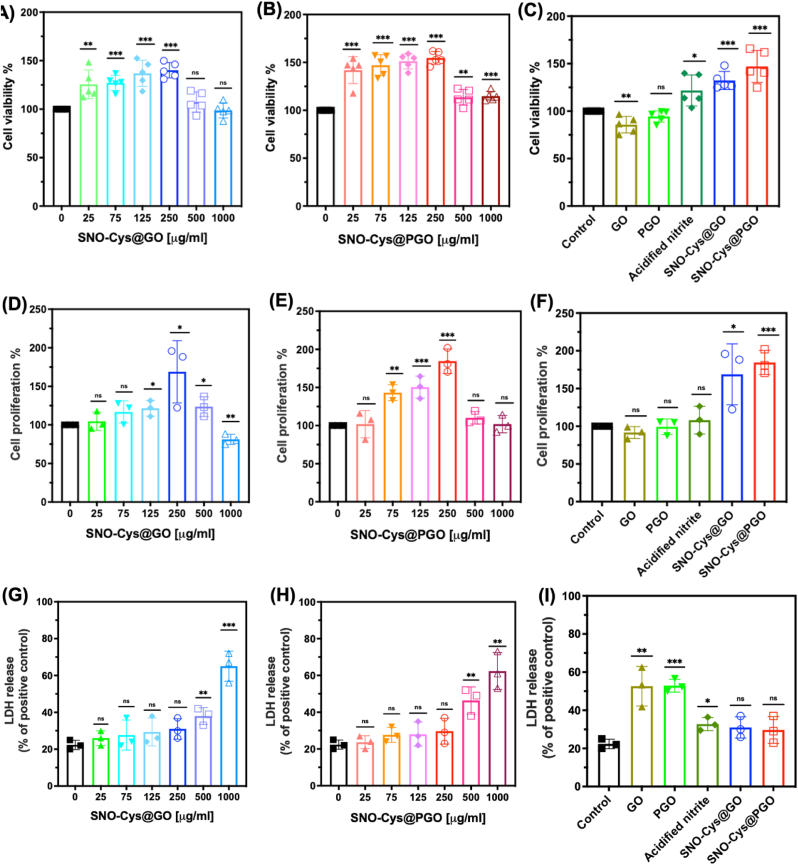
**Effect of graphene formulations on endothelial cell (sEND.1) viability and proliferation*****in vitro*****.(A**–**C)** Cell viability assessed by the MTT assay after a 24-h incubation: (**A**) with increasing concentrations of SNO-Cys@GO, (**B**) with increasing concentrations of SNO-Cys@PGO and **(C)** comparison between formulations of GO, PGO, acidified nitrite, SNO-Cys@GO, and SNO-Cys@PGO at 250 μg/ml. Data are presented from five independent experiments (n = 5) and expressed as a percentage of control cells (assigned 100%). **(D**–**F)** Endothelial cell proliferation evaluated by BrdU uptake after a 24-h incubation: (**D**) with increasing concentrations of SNO-Cys@GO and (**E**) with increasing concentrations of SNO-Cys@PGO and (**F**) comparison between formulations of GO, PGO, acidified nitrite, SNO-Cys@GO, and SNO-Cys@PGO at 250 μg/ml. Data are presented from three independent experiments (n = 3) and expressed as a percentage of control cells (assigned 100%). **(G**–**I)** LDH release from endothelial cells in response to increasing concentrations of (**G**) SNO-Cys@GO and (**H**) SNO-Cys@PGO and (**I**) comparison between formulations of GO, PGO, acidified nitrite, SNO-Cys@GO, and SNO-Cys@PGO at 250 μg/ml. Data are presented from three independent experiments (n = 3) and expressed as LDH release (%) compared to the positive control (lysis solution). All data are shown as mean ± SD with statistical comparison versus control, where ns denotes not significant, *p < 0.05, **p < 0.01, and ***p < 0.001.

Both SNO-Cys@GO and SNO-Cys@PGO demonstrated a dose-dependent enhancement of EC viability and proliferation peaking at 250 μg/ml (Fig. 6A–B and D-E). At this dose, the MTT response was increased by +40% and BrdU by +68 % for SNO-Cys@GO and was higher for SNO-Cys@PGO at +53% and +84 % respectively, demonstrating a strong positive effect to increase proliferation. At higher concentrations, this effect was mostly lost, although viability remained comparable to control cells, suggesting the presence of some toxicity to blunt proliferation. This was confirmed by the LDH assay, where LDH release was only increased in response to SNO-Cys@GO and SNO-Cys@PGO at concentrations of 500 μg/ml and above. Based on these results, we chose the 250 μg/ml dose to compare graphene formulations with and without functionalisation. GO on its own had a small negative effect on cell viability and did not alter proliferation, while PGO did not affect either parameter. However, an increase in LDH release suggests that both unmodified GO and PGO can illicit cellular toxicity. Acidified nitrite on its own increased viability slightly, despite a small increase in LDH release and no effect on proliferation, indicating that conjugation to graphene is necessary to obtain the full pro-proliferative effects observed with SNO-Cys@GO and SNO-Cys@PGO (Fig. 6C, F). These results suggest that functionalised graphene formulations show no measurable cytotoxicity in endothelial cells at concentrations up to 250 μg/ml, potentially due to the ability to release NO.

**Fig. 7:**
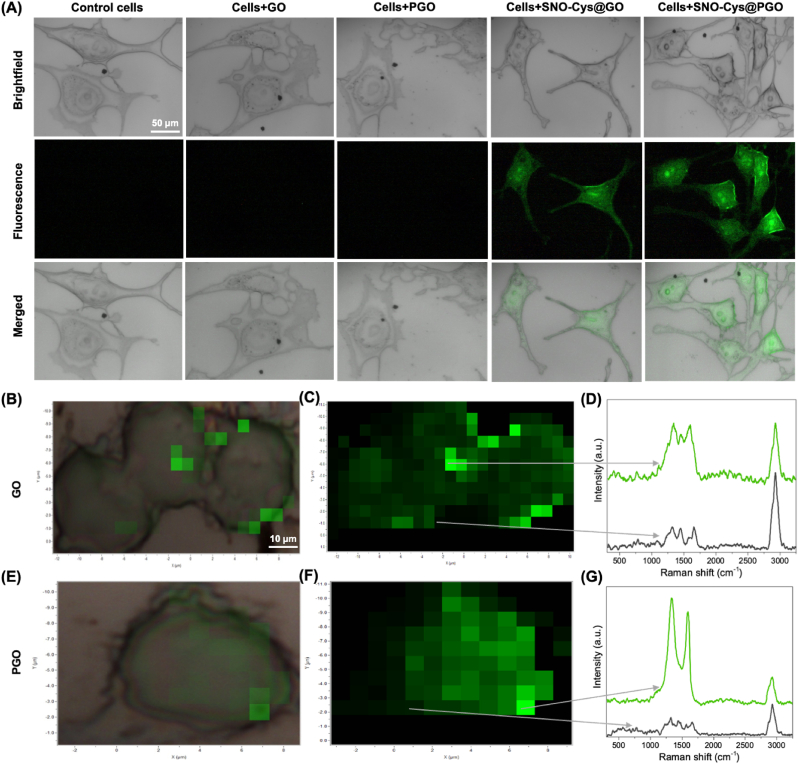
**Cellular uptake of graphene and fluorescent detection of nitric oxide(NO)in sEND.1 endothelial cells*****in vitro*****.(A)** Representative confocal microscopy images of cells treated with l-NAME to block endogenous NO production and incubated with graphene formulations at 250 μg/mL for 1 h. Cells were loaded with the NO-sensitive fluorescent dye DAF-FM DA, which indicates that NO is located intracellularly and only in the presence of SNO-Cys@GO or SNO-Cys@PGO. Scale is identical for all images (bar = 50 μm). **(B**–**G)** Raman micro-spectroscopy of sEND.1 at the single-cell level exposed to 250 μg/ml GO and PGO. Brightfield images overlaid with Raman intensities of GO and PGO in green for co-localisation and visualisation of relative abundance (**B and E**). The corresponding heatmap data is shown in **C and F** (bar = 10 μm applies to all images) from which two contrasting points are labelled in each image: one with a high graphene signal and one with little or no graphene signal. The corresponding Raman spectra for each point are shown (**D and G**) and clearly demonstrate the characteristic signal for graphene indicating cellular uptake of nanoparticles. (For interpretation of the references to colour in this figure legend, the reader is referred to the Web version of this article.)

We further investigated the localisation of NO released from graphene formulations by incubating endothelial cells with a NO-sensitive fluorescent dye (DAF-FM DA) in the presence of *l*-NAME to inhibit endogenous sources of NO production. This indicated that intracellular NO was only detected in the presence of SNO-Cys@GO or SNO-Cys@PGO (Fig. 7A). Raman micro-spectroscopy was used to confirm whether this was due to cellular uptake of GO and PGO. Fig. 7, panels B and **E,** show brightfield images with overlaid pseudo-colour Raman intensities for graphene in endothelial cells treated with 250 μg/ml of GO and PGO respectively. The corresponding heatmaps are shown in Fig. 7, panels C and **F**, with the relative distribution and abundance of GO and PGO indicated in green. Graphene was detected throughout the intracellular space in sEND.1 cells and was not confined to any single localised area, but was absent from the extracellular space. Representative spectra are shown from areas of high and low graphene signal for GO and PGO respectively (Fig. 7, panels D and **G**). The unique and characteristic Raman signal for graphene is clearly identifiable (G-peak at 1600 cm^−1^ and the D-peak at 1350 cm^−1^) and could only arise from the cellular uptake of the graphene nanostructures. The prominent peak at 2940 cm^−1^ is attributable to the lipids of the plasma membrane (symmetric CH_2_ stretch) [42]. Taken together, these results show that GO and PGO are taken up by endothelial cells *in vitro* to release NO intracellularly.

### 2.4 Smooth muscle cell proliferation

**Fig. 8:**
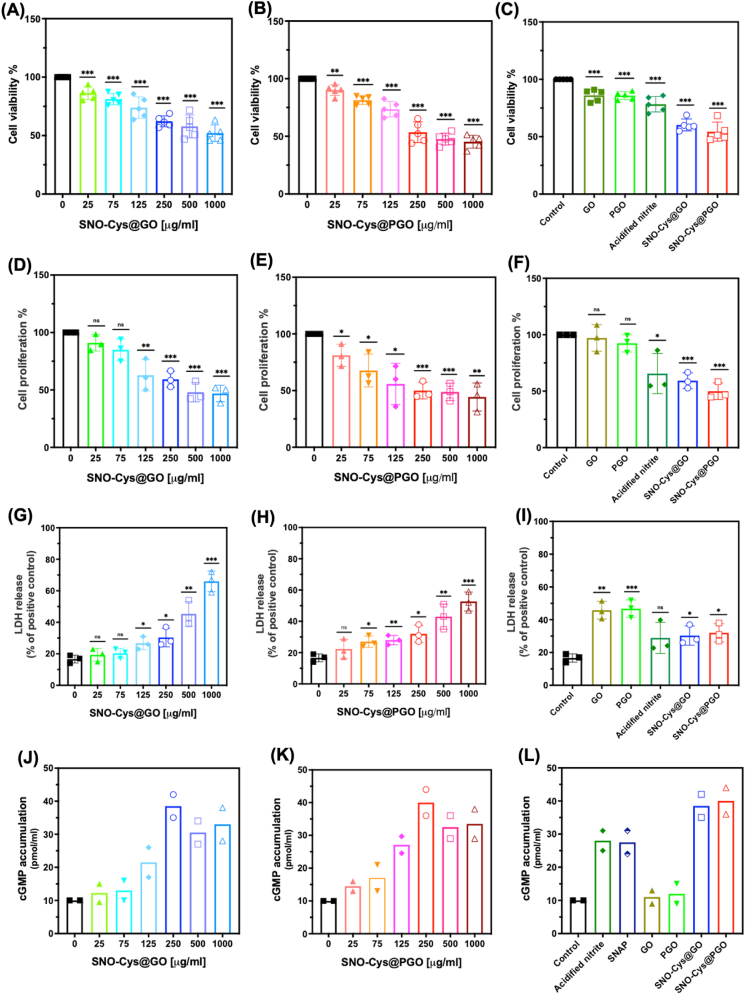
**Effect of graphene formulations on smooth muscle cells (MOVAS)*****in vitro*****.(A, B)** Cell viability assessed by MTT assay after a 24-h incubation with increasing concentrations of: (**A**) SNO-Cys@GO and (**B**) SNO-Cys@PGO. **(C)** Comparison between formulations of GO, PGO, acidified nitrite, SNO-Cys@GO, and SNO-Cys@PGO at 250 μg/ml. Data from five independent experiments (n = 5) are presented, and are expressed as a percentage of control cells (assigned 100%). **(D**–**E)** SMC proliferation evaluated by BrdU uptake after 24-h incubation with increasing concentrations of: (**D**) SNO-Cys@GO and (**E**) SNO-Cys@PGO. (**F**) Comparison between formulations of GO, PGO, acidified nitrite, SNO-Cys@GO, and SNO-Cys@PGO at 250 μg/ml. Data from three independent experiments (n = 3) are presented, and are expressed as a percentage of control cells (assigned 100%). **(G** -**H)** LDH release from SMC in response to increasing concentrations of: (**G**) SNO-Cys@GO and (**H**) SNO-Cys@PGO. (**I**) Comparison between formulations of GO, PGO, acidified nitrite, SNO-Cys@GO, and SNO-Cys@PGO at 250 μg/ml. Data from three independent experiments (n = 3) are presented, and are expressed as LDH release % compared to positive control (lysis solution). All data are shown as mean ± SD with statistical comparison versus control, where ns denotes not significant. *p < 0.05, **p < 0.01, and ***p < 0.001. **(J**–**K)** Cellular accumulation of the intracellular messenger, cGMP, which is released when NO activates guanylate cyclase. SMCs were incubated with graphene formulations and controls for 45 min, with cGMP determined by ELISA, **(L)** Comparison between formulations of GO, PGO, acidified nitrite, SNAP, SNO-Cys@GO, and SNO-Cys@PGO at 250 μg/ml (shown in duplicate).

NO is known to inhibit SMC proliferation, so the same assays of cell viability (MTT), proliferation (BrdU) and toxicity (LDH) were performed in cultured murine vascular SMCs (MOVAS) exposed to various graphene formulations for 24-h. Both SNO-Cys@GO and SNO-Cys@PGO demonstrated a dose-dependent reduction in SMC viability, proliferation across the full range of doses tested (Fig. 8 A-B, D-E, and **G-H**). The effect size and pattern of response is very similar for both parameters, which suggests that the reduction in cell viability predominantly reflects the absence of proliferation, meaning there are fewer cells at 24-h when compared to the control group. However, a small but significant increase in LDH release at lower concentrations of functionalised GO and PGO suggests there is some low-level toxicity, which only becomes pronounced at concentrations of 500 μg/ml and above. Fig. 8C, **F,** and **8I** compare various graphene formulations and acidified nitrite (as a NO source control) at the same concentration of 250 μg/ml. Only those formulations that included NO sources (i.e. acidified nitrite, SNO-Cys@GO and SNO-Cys@PGO) had significantly reduced proliferation, suggesting this is a NO-mediated response that is reflected as lower cell numbers in the viability data. In contrast, both GO and PGO on their own had a significant effect on cell viability and LDH release without affecting proliferation, suggesting a potential toxic effect of non-functionalised graphene to reduce SMC survival. It is worth noting that in a healthy vessel, the dose that SMCs would be exposed to is lower than for ECs, which represent a physical diffusion barrier.

The biological actions of NO, including the inhibition of SMC proliferation, are known to be mediated via activation of soluble guanylate cyclase to generate the intracellular second messenger, cGMP [[43], [44], [45]]. The cellular accumulation of cGMP was therefore measured by ELISA in response to incubation of SMCs with various graphene formulations and controls. The responses for SNO-Cys@GO and SNO-Cys@PGO were both dose-dependent, reaching a maximal response at 250 μg/ml (Fig. 8 J, K). GO and PGO on their own did not elicit an increase in cGMP levels, whereas all sources of NO did i.e. acidified nitrite, SNAP, and particularly SNO-Cys@GO and SNO-Cys@PGO (Fig. 8 L). Thus, the generation of intracellular cGMP demonstrates that NO released from functionalised graphene formulations can activate the canonical NO signalling pathway. Collectively, these results demonstrated the effectiveness of SNO-Cys@GO and SNO-Cys@PGO in enhancing endothelial cell proliferation and inhibiting SMC proliferation via slow and sustained release of NO.

**Fig. 9:**
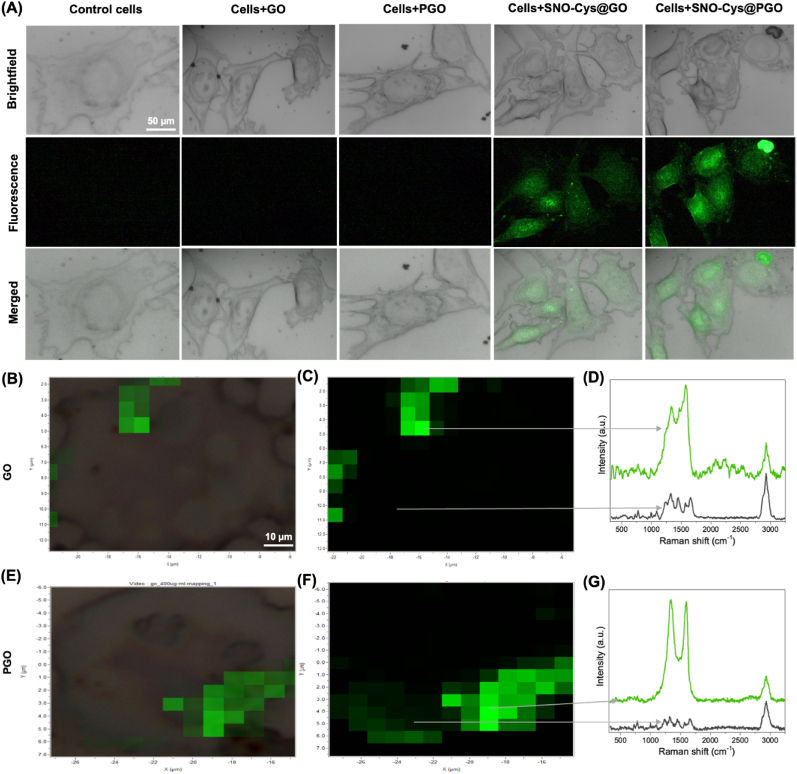
**Cellular uptake of graphene and fluorescent detection of nitric oxide(NO)in MOVAS vascular smooth muscle cells*****in vitro*****.(A)** Representative confocal microscopy images of cells treated with l-NAME to block endogenous NO production and incubated with graphene formulations at 250 μg/mL for 1 h. Cells were loaded with the NO-sensitive fluorescent dye, DAF-FM DA, which indicates that NO is located intracellularly and only in the presence of SNO-Cys@GO or SNO-Cys@PGO. The scale is identical for all images (bar = 50 μm). **(B**–**G)** Raman micro-spectroscopy of MOVAS at the single-cell level exposed to 250 μg/ml GO and PGO. Brightfield images overlaid with Raman intensities of GO and PGO in green for co-localisation and visualisation of relative abundance (**B and E**). The corresponding heatmap data are shown in **C and F** (bar = 10 μm applies to all images) from which two contrasting points are labelled in each image: one with a high graphene signal and one with little or no graphene signal. The corresponding Raman spectra for each point are shown (**D and G**) and clearly demonstrate the characteristic signal for graphene indicating cellular uptake of nanoparticles. (For interpretation of the references to colour in this figure legend, the reader is referred to the Web version of this article.)

As before, the localisation of NO released from graphene formulations was investigated by incubating SMCs with a NO-sensitive fluorescent dye (DAF-FM DA) in the presence of l-NAME. This indicated that NO released from both SNO-Cys@GO and SNO-Cys@PGO was localised intracellularly, suggesting that these graphene formulations are also taken-up into SMCs where they retain NO-releasing activity (Fig. 9A). To further demonstrate the localisation of GO and PGO in SMCs (MOVAS), single-cell Raman micro-spectroscopy was used. Fig. 9B and **E** shows brightfield images with overlaid pseudo-colour Raman intensities for graphene in MOVAS cells treated with 250 μg/ml of GO and PGO respectively. The corresponding heatmaps are shown in Fig. 9C and **F**, with the relative distribution and abundance of GO and PGO in green, indicating that graphene was detected within the intracellular space in MOVAS cells. Representative spectra are shown from areas of high and low graphene signal (Fig. 9D and G), with the unique and characteristic Raman signal for graphene clearly identifiable. Taken together, these results show that GO and PGO are taken up by vascular smooth muscle cells *in vitro* to release NO intracellularly.

### 2.5 Measurements of *N*-nitrosamines

**Fig. 10:**
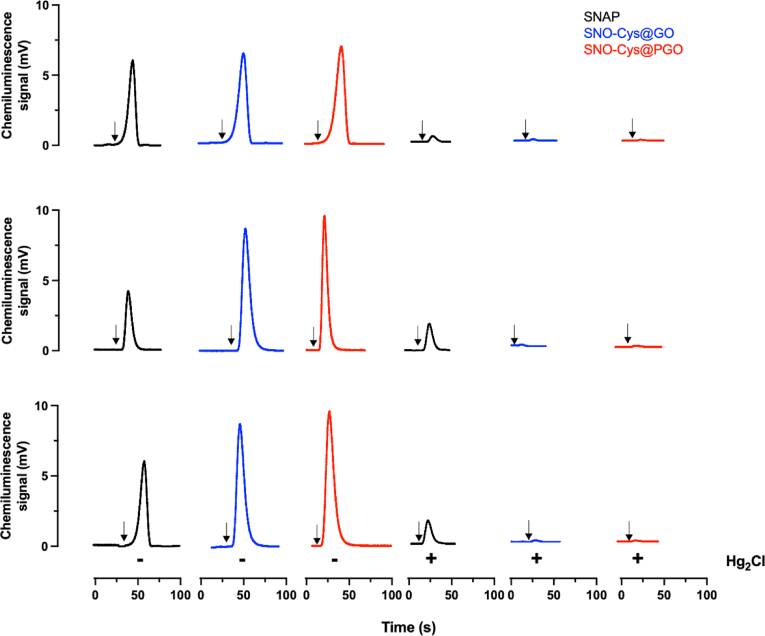
**Representative chemiluminescence time-traces for the detection of*****N*****-nitrosamines (RNNO) from SNAP and NO-releasing graphene formulations (SNO-Cys@GO and SNO-Cys@PGO).** All samples were tested at 250 μg/ml and treated with 5% acidified sulfanilamide to eliminate nitrite. In the absence of HgCl₂ (’-’) the signal represents a combination of RSNO and RNNO, while in the presence of HgCl₂ (’+’), the smaller residual peaks represent RNNO. **(A)** Samples without cells, **(B)** sEND.1 endothelial cells exposed to samples, **(C)** MOVAS smooth muscle cells exposed to samples for 60 min. Arrow (↓) indicates the injection of samples with the x-axis showing time duration since the start of the experimental run (n = 3).

Measurement of *N*-nitrosamines (RNNOs) was performed on all samples, both with and without cells, using ozone-based chemiluminescence. A variety of NO metabolites can be identified based on their reaction chemistry. It has previously been shown that the residual mercury-stable chemiluminescence signal arises from RNNOs which persist after pre-incubating the samples in the reducing system (triiodide, which does not convert nitrate to NO) [46,47]. The mercury resistant signal that has been assigned to RNNOs arises from compounds that have not reacted with sulfanilamide (which reacts with nitrite) and have not been decomposed by the addition of HgCl_2_ (reacts with RSNO) [[47], [48], [49]]. By using this assay, mercury-stable RNNO signals were detected from SNAP both with and without cells (Fig. 10 A-C). SNO-Cys@GO and SNO-Cys@PGO produce RSNO comparable to SNAP, but the residual signals for RNNO were near the limits of detection. These results suggest that both NO releasing graphene formulations (SNO-Cys@GO and SNO-Cys@PGO) create less RNNOs in cells compared to the SNAP control group. There was no detectable signal from cells only (untreated) and unmodified GO and PGO.

Alternative approaches for NO delivery to the heart using NPs have been described in the literature. For example, dendrimers conjugated with SNAP have been reported to reduce ischaemia/reperfusion injury in the isolated, perfused rat heart. NO release from this formulation was highest in the presence of reduced glutathione (GSH), which can decompose SNAP via direct transnitrosation [18,19]. Despite low experimental numbers, this is an important finding, since it establishes proof-of-concept for NO delivery to the myocardium using NPs [18]. Further testing is required *in vivo* using relevant disease models, where the low solubility of dendrimers and the reliance on GSH-dependent NO release may ultimately prove limiting [9]. In our current work, we have described a novel NO delivery system via the reaction of acidified sodium nitrite with thiol groups that had been introduced by cysteamine conjugation to porous graphene oxide nanosheets, thereby generating *S*-nitrosated nanosheets, which we believe may offer some advantages by enabling the precise and controlled release of NO intracellularly. Porous graphene also has desirable properties of high specific surface area and porosity in order to store high amounts of NO sources. Unlike organic nitrates, NO release from *S*-nitrosated graphene nanosheets is not dependent on activation by mitochondrial enzymes, down-regulation of which is one important mechanism in the development of tolerance [50]. Furthermore, common but debilitating adverse effects of nitrate and nitrite-based donor drugs include headache, which is dependent on the vasodilator effect in certain vascular beds. It may therefore be possible to modify biodistribution of graphene delivery systems by tailoring particle size, and surface charge in order to minimise such adverse effects.

Our study has a number of inherent limitations since it represents the basic design and characterisation of novel graphene-based NO-releasing materials. Although we have performed extensive *in vitro* studies to test the interaction with vascular cells, further studies will be required to test these concepts *in vivo*. This will require consideration of stable formulations for administration, pharmacokinetics, interaction with blood components, elimination and ultimately safety and efficacy studies in clinically relevant animal models. Nevertheless, our findings represent an important first step in the development of a new class of graphene-based NO delivery vehicles with therapeutic potential.

### 2.6 Conclusion

In this study, we developed a novel NO delivery system via generating *S*-nitrosated nanosheets through the *S*-nitrosation of thiol groups that had been provided by GO/PGO-conjugated cysteamine. The exchange between functional groups and the corresponding morphologies of the resulting *S*-nitrosocysteamine-functionalised graphene were characterised by XRD, FTIR, Raman spectroscopy, XPS, NMR, and SEM. Functionalised graphene nanostructures were taken up by endothelial and smooth muscle cells *in vitro* to release NO intracellularly. The release of NO enhanced endothelial cell proliferation and inhibited smooth muscle cell growth via activation of canonical NO signalling pathways, which are desirable properties for treating vascular injury. Furthermore, these formulations generated negligible amounts of toxic NO by-products. Collectively, the development of porous graphene as NO delivery vehicles highlights the potential of these formulations as a promising strategy for treating cardiovascular diseases.

## 3 Materials and methods

### 3.1 Chemicals and reagents

Sodium nitrate (NaNO_3_), sulfuric acid (H_2_SO_4_ – 95.0–98.0%), potassium permanganate (KMnO_4_), hydrogen peroxide (H_2_O_2_ – 30 wt%), hydrochloric acid (HCl – 36 wt%), sodium iodide, potassium iodide, iodine, acetic acid (99.5%), sulfanilamide, and MTT assay were purchased from Thermo Scientific, Fisher Scientific, Acros, Nacalai Tesque and Alfa Aesar, respectively. Graphite flakes, *N*-(3-dimethylaminopropyl-*N*′-ethylcarbodiimide) hydrochloride (EDC), *N*-hydroxysuccinimide (NHS), phosphate buffered saline (PBS), potassium bromide (KBr), sodium nitrite (NaNO_2_ – 97%), potassium iodide, vanadium chloride, TEM copper grid, cysteamine, *S*-nitroso-*N*-acetyl-*dl*-penicillamine (SNAP), diethyldithiocarbamic acid sodium salt (DETC), iron (II) sulfate heptahydrate (FeSO_4_·7H_2_O, ≥99%), dichloromethane (CH_2_Cl_2_), mercuric chloride (HgCl_2_), 1 M hydrochloric acid (HCl) and the LDH assay kit (coulorimetric, MAK066) were purchased from Sigma Merck. ATCC medium containing 4 mM l-glutamine, 4500 mg/L glucose, 1 mM sodium pyruvate, and 1500 mg/L sodium bicarbonate was purchased from LGC Limited, UK. cGMP ELISA kits were purchased from Enzo Life Science, UK. BrdU cell proliferation assay kits were purchased from Cell Signalling Technology, UK. DAF-FM DA was purchased from Abcam, UK. All materials were used as received without any further purification unless stated otherwise.

### 3.2 Synthesis of graphene oxide and porous graphene oxide

As we previously reported, exfoliated graphite was converted into exfoliated graphene oxide (GO) flakes using a modified Hummer's approach [22,26,28]. Graphite flakes (2 g) were added to an 800 mL round-bottom flask together with NaNO_3_ (1.5 g) and H_2_SO_4_ (150 mL, 98%). The reaction mixture was combined while being stirred magnetically, and then the flask was submerged in an oil bath. The mixture was then heated to 35 °C before KMnO_4_ (9 g) was added to the flask. The mixture was continuously stirred for 24 h, then 280 ml of H_2_SO_4_ (5%) was added and the temperature was raised to 85–95 °C. The bath was removed after 2 h of stirring the mixture. After that, the flask was allowed to cool to 60 °C. Finally, 15 mL (30 wt%) of H_2_O_2_ was added and the mixture was stirred for another 2 h. To remove any impurities, the product was washed 7–8 times with HCl (3 wt%) and then 4–5 times with distilled water. As-prepared GO was dispersed in water under stirring.

### 3.3 Thiolation of graphene oxide and porous graphene oxide

The resultant exfoliated GO dispersion (100 mL at 1 mg/mL) was combined with 3.4 g of NHS (30.0 mmol) and 5.7 g of EDC (30.0 mmol) in a round-bottom flask in an ice bath. Following 2 h of continuous stirring, 0.40 g of cysteamine (3.52 mmol) was added to the mixture, which was then left to stir at room temperature overnight. The resulting thiol-modified graphene solid (termed Cys@GO and Cys@PGO) was repeatedly washed with distilled water, and dried at 40 °C.

### 3.4 Synthesis of *S*-nitrosocysteamine-functionalised graphene oxide and porous graphene oxide

NO-releasing GO and PGO were prepared via *S*-nitrosation by the addition of sodium nitrite under acidic conditions. Briefly, 50 mg of Cys@GO or Cys@PGO was dissolved in 10 ml (methanol/H_2_O (V/V, 1/1)] in an ice bath, and protected from light under stirring. 1 ml of citric acid (1 M) was added while stirring, and then 1 ml of sodium nitrite (0.5 M) aqueous solution was added dropwise. The mixture was left to react in an ice bath for 60 min while being shielded from light. Following multiple cycles of washing, ultrasonication, centrifugation, and washing with methanol, the SNO-Cys@GO or SNO-Cys@PGO products were vacuum-dried at 25 °C.

### 3.5 Basic characterisation of graphene formulations

GO, PGO and functionalised Cys@GO, SNO-Cys@GO, PGO, Cys@PGO and SNO-Cys@PGO were used for further characterisation. X-ray diffraction (XRD) analysis was conducted using Cu Kα radiation at a voltage of 40 kV and a current of 40 mA. X-ray measurements were collected at a step size of 0.02° (2θ) and a step time of 1 s. Fourier-transform infrared (FTIR) spectroscopy was conducted using a Tensor-27 FTIR spectrometer (Bruker Optics, Champs-sur-Marne, France) in the wavenumber range of 4000–500 cm^−1^. FTIR samples were prepared by mixing the sample with KBr. Raman spectra were obtained using a confocal Raman microscope (LabRAM HR Evolution, Horiba Scientific, UK) equipped with a motorised XYZ stage, an integrated microscope (BX41, Olympus), and a 532-nm laser. With an input laser intensity of 2–4 mW passing through a 25% neutral density (ND) filter, spectra were obtained with a 50 × air objective lens (numerical aperture 0.5), a 0.5 s collection period, and a 300 grooves/mm diffraction grating. For scanning electron microscopy (SEM) analysis, 10 μl of the respective homogenised suspension were placed on a silicon wafer and stored in the fume hood until completely dried. For visualisation of the samples on the Si-wafer using SEM (Sigma 300 V P, 30 μm pinhole aperture, Carl Zeiss Microscopy GmbH, Germany), the SE2 (Everhart–Thornley) and the InLens (In Beam) detectors were used to capture secondary electrons. The working voltage ranged from 1 kV to 3 kV and the working distance varied between 3.8 mm and 6.3 mm. For transmission electron microscopy (TEM), samples were applied to glow-discharged *C*-Flat 1.2/1.3 4C grids. Images were obtained on a 300 kV Titan Krios electron microscope equipped with a Falcon 3 detector and a Cs corrector (CEOS). A drop of each of the as-prepared samples was deposited on a holey carbon copper grid to prepare the TEM samples. VG Multilab 2000 (Al-Kα, γ = 1.486 keV) machine was used to measure X-ray photoelectron spectroscopy (XPS) of samples in the high vacuum environment. Samples were spread on the Cu foil and were measured on the different spots to identify the isotropy of the sample. Final XPS spectra were analysed using Casa-XPS software. ^13^C Solid state NMR spectra were obtained at 100.5 MHz (9.4 T) on a Bruker Avance IIIHD spectrometer. Samples were packed in 4 mm optical density zirconia rotors and spectra were acquired using a MAS rate of 10 and 14 kHz using single pulse excitation and SPINAL-64 proton decoupling. Depending on the sample volume, 2000 to 25,000 transients were acquired using a 2.5 μs^13^C 90⁰ pulse, an acquisition time of 50 ms and a recycle delay of 10 s. All ^13^C spectra were referenced to glycine (the α-carbon resonance was taken to be at *δ* = 176.03 ppm on a scale where *δ*(TMS) = 0) as a secondary reference.

### 3.6 Measurement of NO release

The NO released from graphene derivatives was purged from the test solution with helium gas and detected by a chemiluminescence NO analyser (NOA) (Sievers 280, Boulder, CO). An aliquot of each sample was introduced directly into the solution in a gas-tight syringe through the side injection port of the NOA vessel under a constant flow/purge of helium gas and protected from exposure to light. A baseline signal was acquired for several minutes, and then an aqueous sample solution (20 μM) was injected into the cell. After the sample injection, each experiment was allowed to continue until NO detection returned to the baseline level. In all cases, a known gas phase NO concentration (ppb/ppm) was used to determine total NO release (mol) using an instrument-specific calibration constant obtained from the reduction of NaNO_2_. Briefly, the amount of NO evolved from the test solution was calculated based on the calibration curves of the NOA, which were regularly obtained by plotting the integrated NOA signal (M) during calibration vs. the introduced amount (moles) of NaNO_2_ into the system via nitrite reduction in an acidified vanadium chloride solution. Raw data was processed using Microsoft Excel 2010. The system was continuously sampled with helium gas.

Real-time NO generation was monitored by a free radical analyser (TBR4100, World Precision Instruments) equipped with an NO-sensitive electrode (ISO–NOP, World Precision Instruments). The NO electrode was first polarized and calibrated following the instruction manual. In detail, the probe was immersed in 10 mL 0.1 M H_2_SO_4_/0.1 M potassium iodide solution in a glass vial to obtain a stable current baseline. Incremental volumes of 25 μM NaNO_2_ solution were added to the mixture to generate a series of NO concentrations for the calibration curve. The generated NO concentrations were calculated according to the amount of NaNO_2_ input as the conversion of NaNO_2_ to NO was stoichiometrically 1:1. To detect the concentration of NO produced from SNO-Cys@GO, and SNO-Cys@PGO (250 µg/mL) in PBS buffer (pH 7.4), changes in current response were recorded in a real-time fashion and produced NO was calculated from the obtained calibration curve. Glass vials were covered by aluminium foil to prevent light exposure, and kept at 37 °C on a hot plate with constant stirring during the NO measurement.

NO release from samples was also detected using electron paramagnetic resonance (EPR) spectroscopy. As described in previous studies [[51], [52], [53]], DETC_2_Fe was used for NO trapping. NaDETC (250 mM, 5 ml) and iron (II) sulfate (FeSO_4_·7H_2_O, 50 mM, 5 ml) were separately dissolved in degassed Milli-Q water. These were immediately mixed in dichloromethane (CH_2_Cl_2_, 10 ml) to prepare a DETC_2_Fe solution. Graphene formulations at the concentrations of 250 μg/ml were incubated with the spin trap complex solution for 30 min. An aliquot (20 μL) of the resulting solution was put into an EPR quartz tube and was examined using X-band EPR spectroscopy. The EPR spectrum displays three lines revealing the formation of NO-DETC_2_Fe complex. EPR spectra were obtained using a continuous wave (CW) homodyne microwave bridge and TE_011_ resonator. The Bruker BioSpin EMXmicro spectrometer was utilised with a Premium X-band (9.1–9.9 GHz) source. The microwave frequency was set at 9.877 GHz, with a power of 10 mW and a field modulation of 0.3 mT. A super high-quality Bruker BioSpin SHQE-W1 in a 0.6 T electromagnet was used as the resonator. All measurements were performed at room temperature with 1.2 mm ID clear fused quartz capillaries and nitrogen gas purge. The amplitudes of the signals were normalized by the experimental parameters, and the samples filled the vertical height of the resonator. The nitrosyl iron signal was located on the slope of a notable ferromagnetic signal of iron oxides at 50 mM Fe(II)SO4 and 250 mM DETC.

### 3.7 Cell viability, proliferation and toxicity assays

The endothelial cell line sENd.1 cells were cultured in Dulbecco's Modified Eagle's Medium (DMEM, Sigma Aldrich) supplemented with 1.5 g/L sodium bicarbonate, 10% fetal bovine serum (Sigma Aldrich), 100 U/mL of penicillin, and 100 μg/mL of streptomycin (Sigma Aldrich). While smooth muscle cells, MOVAS, were cultured in ATCC DMEM supplemented with 4 mM l-glutamine, 4500 mg/L glucose, 1 mM sodium pyruvate, and 1500 mg/L sodium bicarbonate. Cells were grown under standard incubation conditions to reach 70–80 % confluence. The cytotoxicity studies were performed in 96*-*well tissue culture plates (Merck, Sigma Aldrich, UK). The cells were dissociated and plated in 96*-*well plates at 1 × 10^6^ cells per well while cells for imaging were incubated in glass bottom dishes at a density of 1 × 10^6^ cells and incubated for 24 h prior to the experiments. After 24 h incubation, cells were again incubated after treatment with and without 25, 75, 125, 250, 500, 1000 μg/mL of SNO-Cys@GO or SNO-Cys@PGO or 250 μg/mL of acidified nitrite, GO, PGO and SNO-Cys@GO and SNO-Cys@PGO for 24 h. Stock solutions were then further diluted with Milli-Q water to attain the concentrations. Cell viability after treatments was evaluated using MTT assay (Sigma Aldrich, UK) according to the manufacturer's instructions. Briefly, after 24 h incubation of cells with different formulations, culture media was removed, and the cells were then washed twice with PBS. 100 μL of 10% v/v MTT solution (5 mg/mL in PBS) in culture media was added and incubated at 37 °C for 3 h The medium was removed and 100 μL of 20% (w/v) sodium dodecyl sulfate solution and 0.6% (v/v) 37% HCl in dimethyl sulfoxide was added to each well. The concentration of solution was evaluated by optical density (OD) measurements using a xMark™ microplate absorbance spectrophotometer (Bio-Rad) at a wavelength of 570 nm (reference wavelength at 630 nm). The results were expressed as percentage cell viability. Five independent experiments were performed for each sample and all measurements were performed in triplicate.

The proliferation of cells exposed to formulations (GO, PGO, SNO-Cys@GO, SNO-Cys@PGO, acidified nitrite) was analysed using the BrdU colorimetric assay kit (Cell signalling, UK). Cells (sEND.1 or MOVAS) were seeded in 96 well plates at 1 × 10^6^ cells/well/0.1 ml medium followed by overnight incubation. Cells were exposed to 25, 75, 125, 250, 500, 1000 μg/mL of SNO-Cys@GO or SNO-Cys@PGO or 250 μg/mL of acidified nitrite, GO, PGO and SNO-Cys@GO and SNO-Cys@PGO for 24 h. After 24 h of incubation, the BrdU assay was performed according to the manufacturer's instructions and analysed at 450 nm.

For the lactate dehydrogenase (LDH) assay, cells (sEND.1 or MOVAS) were seeded in 96 well plates at 1 × 10^6^ cells/well/0.1 ml medium followed by overnight incubation. Cells were exposed to 25, 75, 125, 250, 500, 1000 μg/mL of SNO-Cys@GO or SNO-Cys@PGO or 250 μg/mL of acidified nitrite, GO, PGO and SNO-Cys@GO and SNO-Cys@PGO for 24 h. After removing the media from each well, it was centrifuged for 5 min at 1200 rpm. A new 96-well plate was filled with 50 μL of the medium supernatant and the LDH assay reagent, and the plate was incubated for 45 min. The absorbance was measured at 490 nm. To prepare a positive control, 10 μL of lysis solution was added to the control cells 45 min prior to centrifugation. The data were expressed as LDH release (%) compared to the positive control (lysis solution).

### 3.8 cGMP analysis

A cGMP ELISA kit (Enzo Life Sciences, New York, USA) was used to measure the accumulation of cGMP in SMCs in accordance with the manufacturer's instructions. Samples (25, 75, 125, 250, 500, 1000 μg/mL of SNO-Cys@GO or SNO-Cys@PGO or 250 μg/mL of acidified nitrite, SNAP, GO, PGO and SNO-Cys@GO and SNO-Cys@PGO) were added to the plate provided by the supplier. The wells were filled with the conjugate and the antibody. Following a 45-min incubation period at room temperature, the contents were taken out and the wells were given three washings. Each well received a pNpp substrate solution, which was then left to incubate for an additional hour at room temperature. After adding the stop solution, the absorbances at 405 nm were measured immediately. Interpolation was used to determine the cGMP concentrations.

### 3.9 Cell imaging

Cells were cultured overnight on IBIDI coverslips (in 3-wells) at 37 °C in a 5% CO_2_ atmosphere. Cells were treated with *l*-NAME (5 μM) for 60 min and then were treated under different conditions: (1) without further treatment, (2) with GO (250 μg/ml), (3) PGO (250 μg/ml), (4) SNO-Cys@GO 250 μg/ml), (5) SNO-Cys@PGO (250 μg/ml) and were incubated for 60 min The medium was removed and gently washed with PBS. 5 μM DAF-FM DA dye (dissolved in 50 μl PBS) was added to each well and cells were incubated for 15 min. The cells were gently washed with PBS once and fixed with a 4% paraformaldehyde solution. The cells were examined on a Zeiss Meta 510 LSM confocal microscope. The data were analysed by ImageJ software.

### 3.10 Single-cell Raman mapping

Raman micro-spectroscopic mapping was used to demonstrate the presence of GO and PGO in cells. Prior to Raman acquisition, cells (sEND.1 and MOVAS) were washed through centrifugation. Cells were then diluted in deionised water. 2 μl of cells was then placed onto an aluminium-coated Raman slide. Five duplicates were used to count all the cells. 1 μl of each graphene sample (GO and PGO at a concentration of 250 μg/ml) were mounted on the Raman slide and incubated for 30 min and allowed to air dry at room temperature. A confocal Raman microscope (LabRAM HR Evolution, Horiba Scientific, UK) with an integrated microscope (BX41, Olympus), a motorised XYZ stage, and a 532-nm neodymium-yttrium aluminium garnet laser was used to capture Raman spectra. Spectra were collected using a 50 × air objective lens (numerical aperture 0.5), a 0.5-s collection time, and a 300 grooves/mm diffraction grating for an input laser power of 80 mW passing through a 25% neutral density (ND) filter. The laser power on a single cell was about 4.2 mW. In each condition, >10 cells were randomly selected and scanned at the *x*- and *y*-axis to obtain single-cell maps. 200 sample points were included in each map throughout the mapping process, which involved mapping an area of 2–4 cells and acquiring a total of 150 spectra with a spatial resolution of ∼1.5 μm between each spectrum. An exposure time of 2 s was applied to each spectrum. The positions (exact wavenumber) of graphene and the heatmap intensity are based on the integrated intensity of this region. The proportions of cell areas with and without GO/PGO were calculated; statistical analysis was performed by Student's t-test. All analyses and visualisation were performed using R (version 4.0.0, R Foundation for Statistical Computing, Vienna, Austria). The allocated colours were used to create heatmaps that depicted the spatial distribution and abundance of each component.

### 3.11 Measurement of nitrosamines

Measurement of *N*-nitrosamines (RNNOs) was performed on all samples, both with and without cells, using ozone-based chemiluminescence. To measure RSNOs, tri-iodide was used in the purge vessel at 60 °C [49]. 2 g of potassium iodide (KI) and 1 g of iodine were dissolved in 40 ml of ultrapure water to prepare the tri-iodide solution. The solution was stirred for 30 min. Then, 140 ml of acetic acid was added. All samples were tested at a concentration of 250 μg/ml. Samples treated with 5% acidified sulfanilamide in 1 M HCl to eliminate nitrite [49,54]. In the absence of HgCl₂, the signal represents a combination of RSNOs and RNNOs, while in the presence of HgCl₂, the smaller residual peaks represent RNNO as suggested by previous studies [47,48]. The samples were treated with 5% acidified sulfanilamide for 3 min. For RNNO measurements, the samples were further treated with 0.2% HgCl₂ for 3 min. An aliquot of 100 μl of each pre-incubated sample was introduced directly into the solution in a gas-tight syringe through the side injection port of the NOA vessel under a constant flow/purge of helium gas and protected from exposure to light and detected by a chemiluminescence NO analyser (NOA) (Sievers 280, Boulder, CO). Tri-iodide solution was changed following the measurements of each individual sample. To measure the production of RNNO in cells (sEND.1 and MOVAS), all samples including SNAP and NO-releasing graphene formulations (SNO-Cys@GO and SNO-Cys@PGO) were added to cells and were incubated for at least 60 min at room temperature. Samples were then centrifuged at 10,000×*g* for 10 min and the supernatant was used for chemiluminescence analysis as described above. The incubation time of 60 min was chosen based on the NO release profile of graphene formulations because they release most of the NO in first 60 min. Based on NO release profiles (detected by electrode sensor, ozone-based chemiluminescence and EPR) and cell proliferation experiments, we chose the 250 μg/ml concentration for all formulations to compare the effects on RNNO generation.

### 3.12 Statistical analysis

All statistical comparisons were calculated using GraphPad Prism 9 (GraphPad Software). Comparisons between the different formulations at given time points were made using one-way ANOVA with Dunnett's post-hoc test for multiple comparisons with the control group.

## Funding

This work was supported by a British Heart Foundation (BHF) Fellowship (FS/ATA/21/20015) to TAT. Work in the authors’ laboratory is funded by BHF programme grant (RG/18/12/34040) to CL. W.K.M. is supported by the Department of Chemistry and grants EPSRC (to CAESR: EP/V036408/1; EP/L011972/1) and John Fell Fund (0007019).

## CRediT authorship contribution statement

**Tanveer A. Tabish:** Conceptualization, Data curation, Formal analysis, Funding acquisition, Investigation, Methodology, Project administration, Resources, Software, Supervision, Validation, Visualization, Writing – original draft, Writing – review & editing. **Mian Zahid Hussain:** Data curation, Investigation, Resources, Writing – review & editing. **Sevasti Zervou:** Methodology. **William K. Myers:** Methodology, Resources, Writing – review & editing. **Weiming Tu:** Investigation. **Jiabao Xu:** Data curation, Investigation, Writing – review & editing. **Irina Beer:** Data curation, Resources. **Wei E. Huang:** Resources, Writing – review & editing. **Rona Chandrawati:** Formal analysis, Writing – review & editing. **Mark J. Crabtree:** Resources, Validation. **Paul G. Winyard:** Formal analysis, Funding acquisition, Investigation, Supervision, Validation, Writing – review & editing. **Craig A. Lygate:** Formal analysis, Funding acquisition, Investigation, Resources, Supervision, Validation, Writing – review & editing.

## Declaration of competing interest

Authors declare no conflict of interest.
