# Supplementary material for: S-nitrosocysteamine-functionalised porous graphene oxide nanosheets as nitric oxide delivery vehicles for cardiovascular applications
Source: Redox Biol. 2024 Apr 4;72:103144. doi: 10.1016/j.redox.2024.103144 (PMC11026843; doi:10.1016/j.redox.2024.103144)
Supplement: Multimedia component 1 [file mmc1.docx]

**Supplementary information**

***S*-Nitrosocysteamine-functionalised porous graphene oxide nanosheets as nitric oxide delivery vehicles for cardiovascular applications**

Tanveer A. Tabish^1^, Mian Zahid Hussain^2^, Sevasti Zervou^1^, William K. Myers^3^, Weiming Tu^4^, Jiabao Xu^4,5^, Irina Beer^6^, Wei E. Huang^4^, Rona Chandrawati^7^, Mark J. Crabtree^1,8^, Paul G. Winyard^9^, Craig A. Lygate^1^

^1^ Division of Cardiovascular Medicine, Radcliffe Department of Medicine, British Heart Foundation (BHF) Centre of Research Excellence, University of Oxford, Headington, Oxford OX3 7BN, United Kingdom

^2^ School of Natural Sciences and Catalysis Research Centre, Department of Chemistry, Chair of Inorganic and Metal-Organic Chemistry, Technical University of Munich (TUM), Lichtenbergstraße 4, 85748 Garching, Germany

^3^ Centre for Advanced Electron Spin Resonance (CAESR), Inorganic Chemistry Laboratory, Department of Chemistry, University of Oxford, Oxford, OX1 3QR, United Kingdom

^4^ Department of Engineering Science, University of Oxford, Oxford OX1 3PJ, United Kingdom

^5^ James Watt School of Engineering, University of Glasgow, Glasgow, G12 8QQ, United Kingdom

^6^ Institute of Water Chemistry, Chair of Analytical Chemistry and Water Chemistry, Technical University of Munich, Lichtenbergstraße 4, 85748 Garching, Germany

^7^ School of Chemical Engineering and Australian Centre for Nanomedicine (ACN), The University of New South Wales (UNSW), Sydney, NSW, 2052, Australia

^8^ Department of Biochemical Sciences, School of Biosciences and Medicine, University of Surrey, Guildford, GU2 7XH, United Kingdom

^9^ University of Exeter Medical School, Faculty of Health and Life Sciences, University of Exeter, Exeter EX1 2LU, United Kingdom

Corresponding author

Email address: [tanveer.tabish@cardiov.ox.ac.uk](mailto:tanveer.tabish@cardiov.ox.ac.uk) (T. A. Tabish)


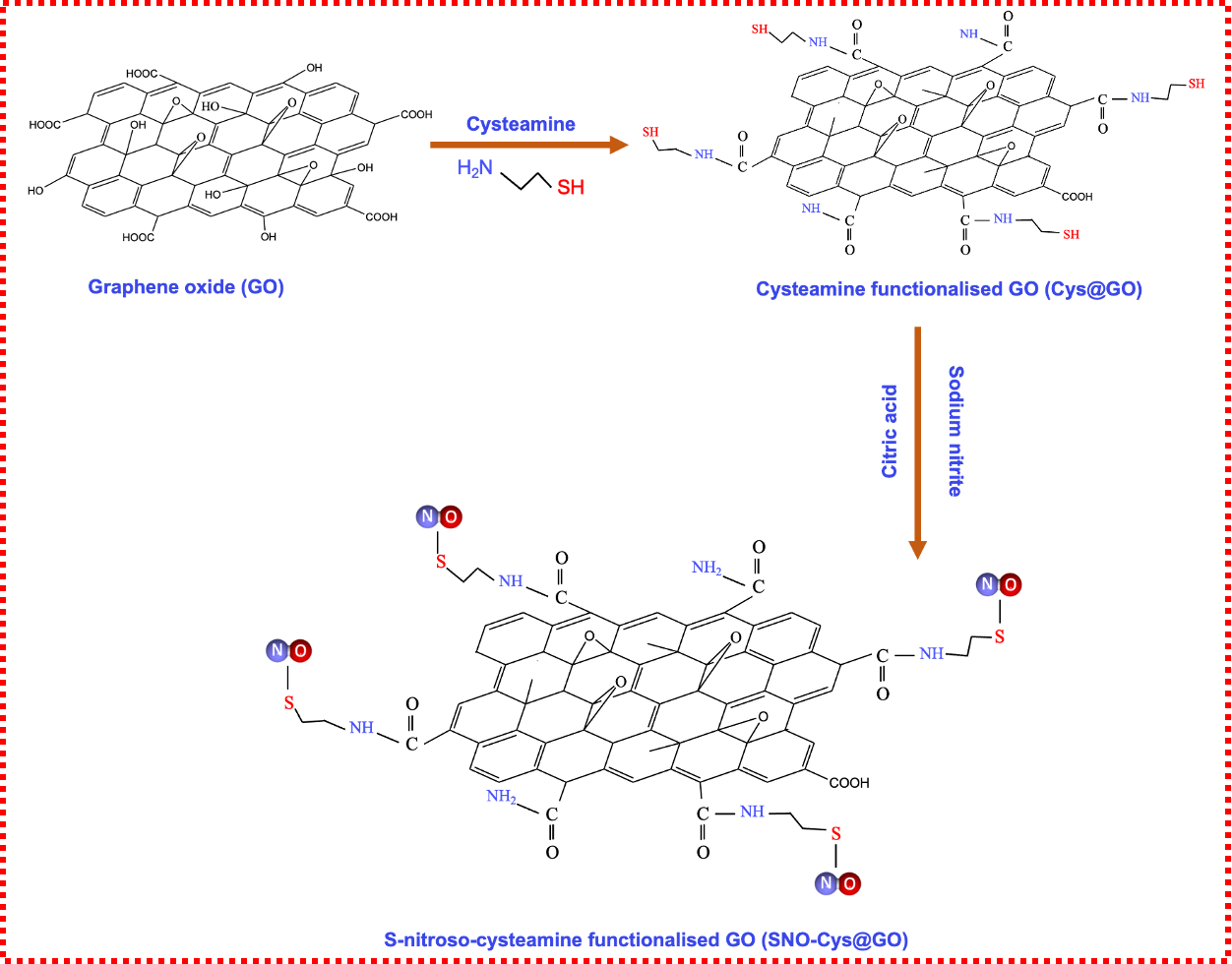


SI Figure 1: A schematic representation of the crosslinking of cysteamine to graphene oxide (GO) followed by the conjugation of S-nitroso to cysteamine functionalised GO to form S-nitroso-cysteamine functionalised GO (termed as SNO-Cys@GO).

SI Figure 2: XRD patterns of acidified sodium nitrite and cysteamine.

SI Figure 3: Raman spectra of acidified sodium nitrite and cysteamine.

SI Figure 4: FTIR of acidified sodium nitrite and cysteamine.


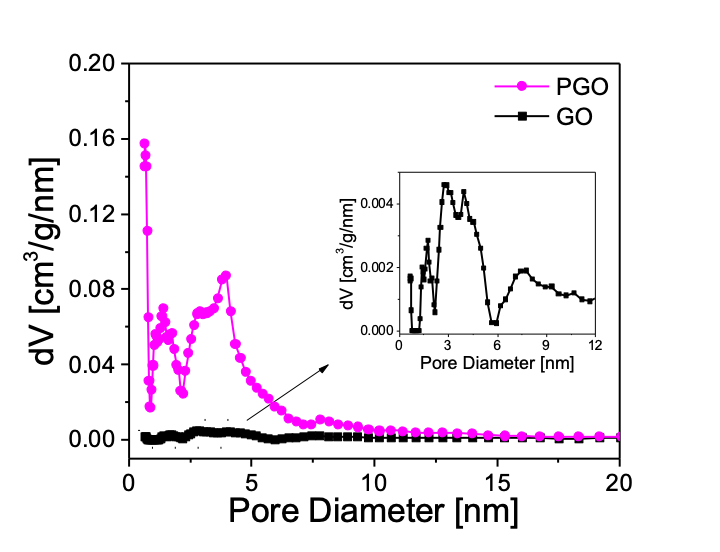


SI Figure 5: The pore size distribution of PGO after activation with HNO_3_, which was characterised by NLDFT method. Compared to the GO (inset), the PGO exhibits the manifold increase in micro/mesopores between 1-15nm.

^
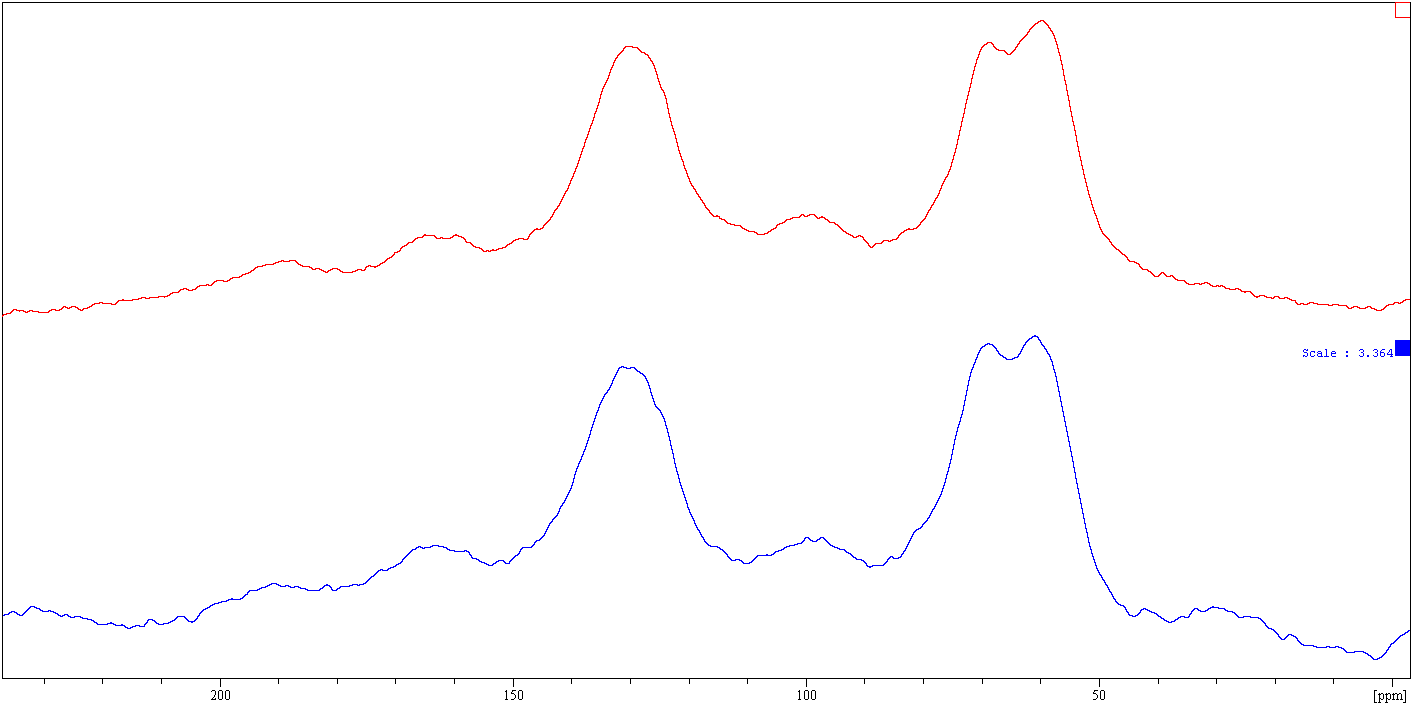
^

SI Figure 6: ^13^C SSNMR spectrum of sample GO measured at 14(top) and 10kHz (bottom) MAS rate.


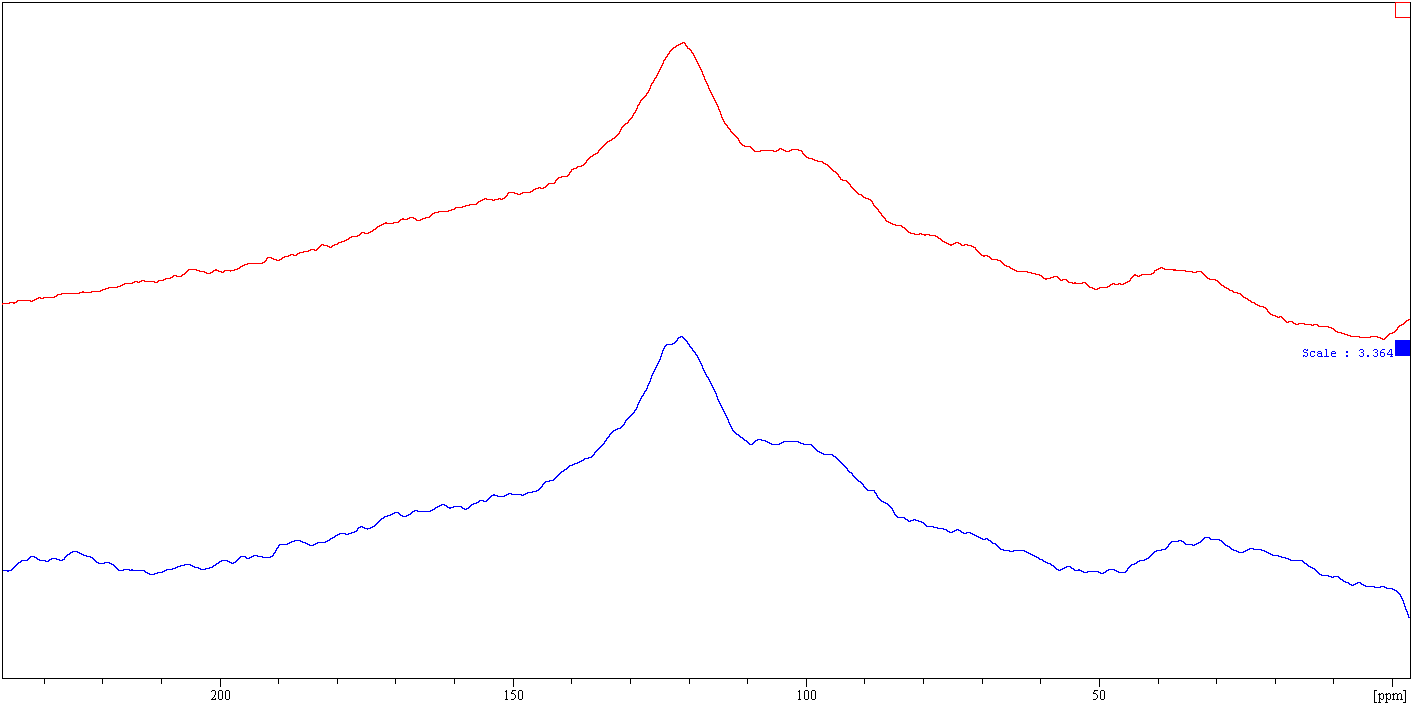


SI Figure 7: ^13^C SSNMR spectrum of sample SNO-Cys@GO measured at 14(top) and 10kHz (bottom) MAS rate.


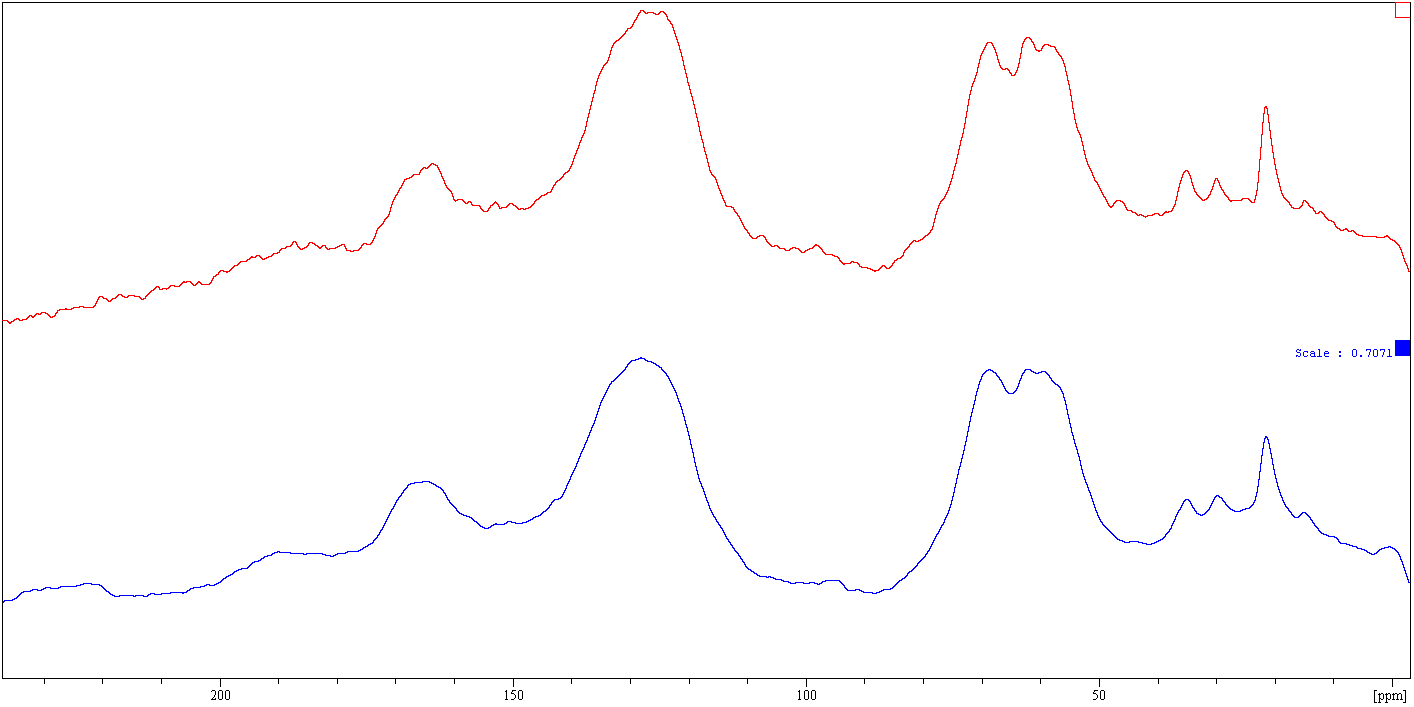


SI Figure 8: ^13^C SSNMR spectrum of sample PGO measured at 14 (top) and 10kHz (bottom)MAS rate.


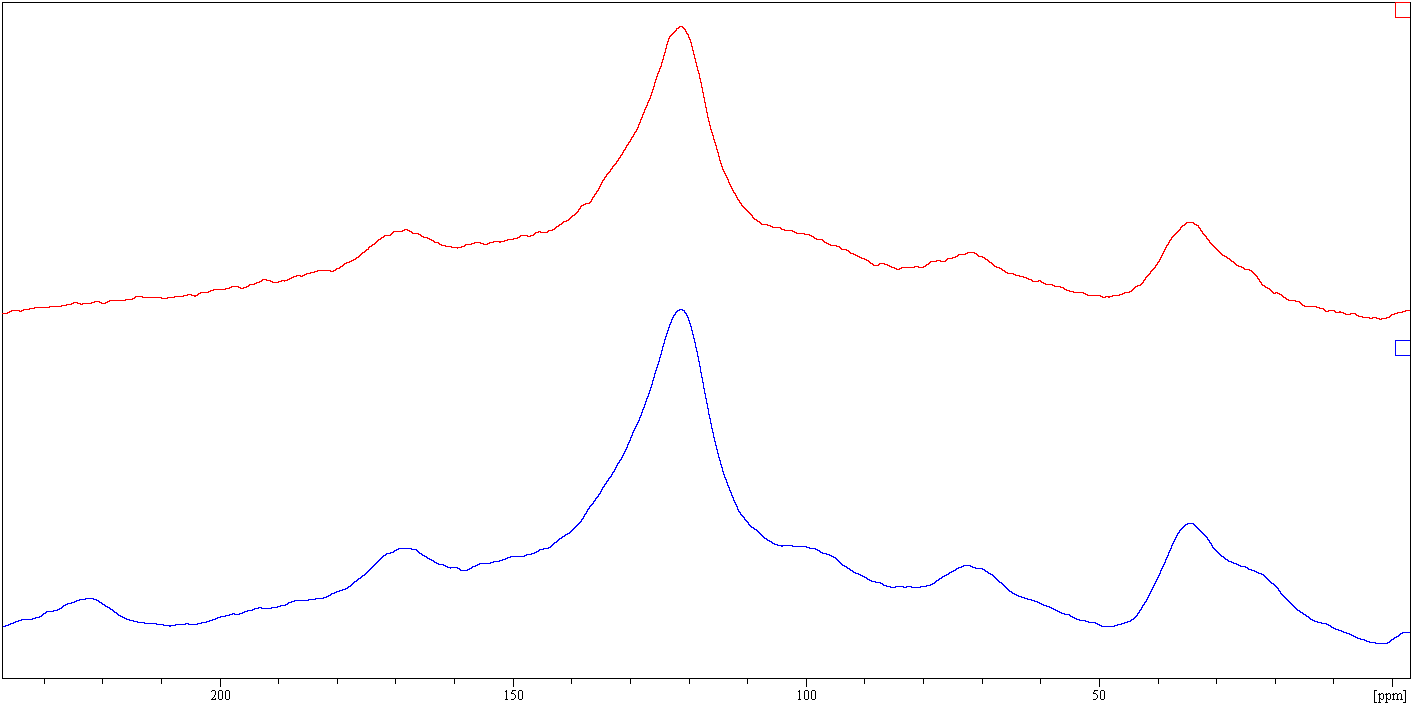


SI Figure 9: ^13^C SSNMR spectrum of sample SNO-Cys@PGO measured at 14 (top) and 10kHz (bottom) MAS rate.
